# Implementation of structured reporting in clinical routine: a review of 7 years of institutional experience

**DOI:** 10.1186/s13244-023-01408-7

**Published:** 2023-04-11

**Authors:** Tobias Jorg, Moritz C. Halfmann, Gordon Arnhold, Daniel Pinto dos Santos, Roman Kloeckner, Christoph Düber, Peter Mildenberger, Florian Jungmann, Lukas Müller

**Affiliations:** 1 Department of Diagnostic and Interventional Radiology, University Medical Center of the Johannes Gutenberg-University Mainz, Langenbeckst. 1, 55131 Mainz, Germany; 2 Department of Radiology, University Hospital of Cologne, Cologne, Germany; 3 Department of Radiology, University Hospital of Frankfurt, Frankfurt, Germany; 4 Institute of Interventional Radiology, University Hospital Schleswig-Holstein - Campus Lübeck, Lübeck, Germany

**Keywords:** Structured reporting, Free-text reporting, Radiology workflow

## Abstract

**Background:**

To evaluate the implementation process of structured reporting (SR) in a tertiary care institution over a period of 7 years.

**Methods:**

We analysed the content of our image database from January 2016 to December 2022 and compared the numbers of structured reports and free-text reports. For the ten most common SR templates, usage proportions were calculated on a quarterly basis. Annual modality-specific SR usage was calculated for ultrasound, CT, and MRI. During the implementation process, we surveyed radiologists and clinical referring physicians concerning their views on reporting in radiology.

**Results:**

As of December 2022, our reporting platform contained more than 22,000 structured reports. Use of the ten most common SR templates increased markedly since their implementation, leading to a mean SR usage of 77% in Q4 2022. The highest percentages of SR usage were shown for trauma CT, focussed assessment with ultrasound for trauma (FAST), and prostate MRI: 97%, 95%, and 92%, respectively, in 2022. Overall modality-specific SR usage was 17% for ultrasound, 13% for CT, and 6% for MRI in 2022. Both radiologists and referring physicians were more satisfied with structured reports and rated SR better than free-text reporting (FTR) on various attributes.

**Conclusions:**

The increasing SR usage during the period under review and the positive attitude towards SR among both radiologists and clinical referrers show that SR can be successfully implemented. We therefore encourage others to take this step in order to benefit from the advantages of SR.

**Key points:**

1. Structured reporting usage increased markedly since its implementation at our institution in 2016.
2. Mean usage for the ten most popular structured reporting templates was 77% in 2022.
3. Both radiologists and referring physicians preferred structured reports over free-text reports.
4. Our data shows that structured reporting can be successfully implemented.
5. We strongly encourage others to implement structured reporting at their institutions.

## Background

Structured reporting (SR) in radiology is often viewed as the favourable form of reporting and is highly recommended by the professional societies [1, 2]. There are numerous advantages of structured reports compared with conventional free-text reports, including better comparability, better readability, and more detailed content [3, 4]. These have been proven for the reporting of various examination types in different imaging modalities over the past years, including trauma CT, prostate MRI, and pulmonary embolism CT [3, 5, 6].

The term SR refers to an IT-based method of importing and arranging medical content in the radiological report and should not be confused with standardised reporting [7]. SR enables the automated acquisition of large quantities of structured data, which can be used for secondary use of data [8]. Secondary use of data can facilitate epidemiologic research and even enable the development of new disease-scoring systems [9]. Structured reports can be further augmented by interactive multimedia elements, such as graphical visual aids or hyperlinks from the text to referenced images [10].

Despite its potential, SR has not become the main form of radiology reporting. Most publications on SR start off with sentences like *“Most radiology reports are written as free text and lack structure”* or *“Traditionally, reports are written as free text”* [3, 11]. Nevertheless, actual use proportions of SR in clinical routine are unclear. Evidence for the implementation of SR is scarce [12].

This might be attributed to the drawbacks of SR: reporting complex cases can be time-consuming in template-based structured reports [13]. Incidental findings different from the suspected diagnosis do not fit well in SR templates. Additionally, most templates consist of checkboxes and drop-down menus. Filling in those templates by using a mouse and keyboard instead of speech recognition, which is commonly used for free-text reporting (FTR), carries the risk of distraction from the image study [14].

The clinical implementation of SR requires relevant changes to the individual radiologist’s workflow and requires great institutional effort [15]. Obstacles in the way of success are IT workflow barriers such as poor integration of SR templates into the radiology information system (RIS) or limited interoperability of healthcare IT systems, as well as personal aversion to SR among radiologists themselves [15, 16]. The Integrating the Healthcare Enterprise Management of Radiology Report Templates (IHE MRRT) profile provides a standardised description of a technical implementation and is freely available [17]. A detailed guide to the clinical implementation of SR, which addresses mainly technical and organisational aspects, is available [18].

In recent years, an extensive amount of research has been conducted in the field of SR. The focus has mostly been on model evaluations of its advantages over FTR and the potential benefits of the acquisition of large standardised datasets, while evidence of its acceptance in real clinical practice remains sparse [12, 19]. Thus, the purpose of this study was to gain more information on the actual status of SR usage in clinical routine. Therefore, we evaluated the implementation process at our institution over a 7-year period from its beginning in 2016. We measured changes in the proportions of structured reports and free-text reports for different examinations and imaging modalities. During the implementation process, a survey among radiologists and referring physicians was conducted to learn their opinions on the different forms of reporting.

## Methods

**Fig. 1:**
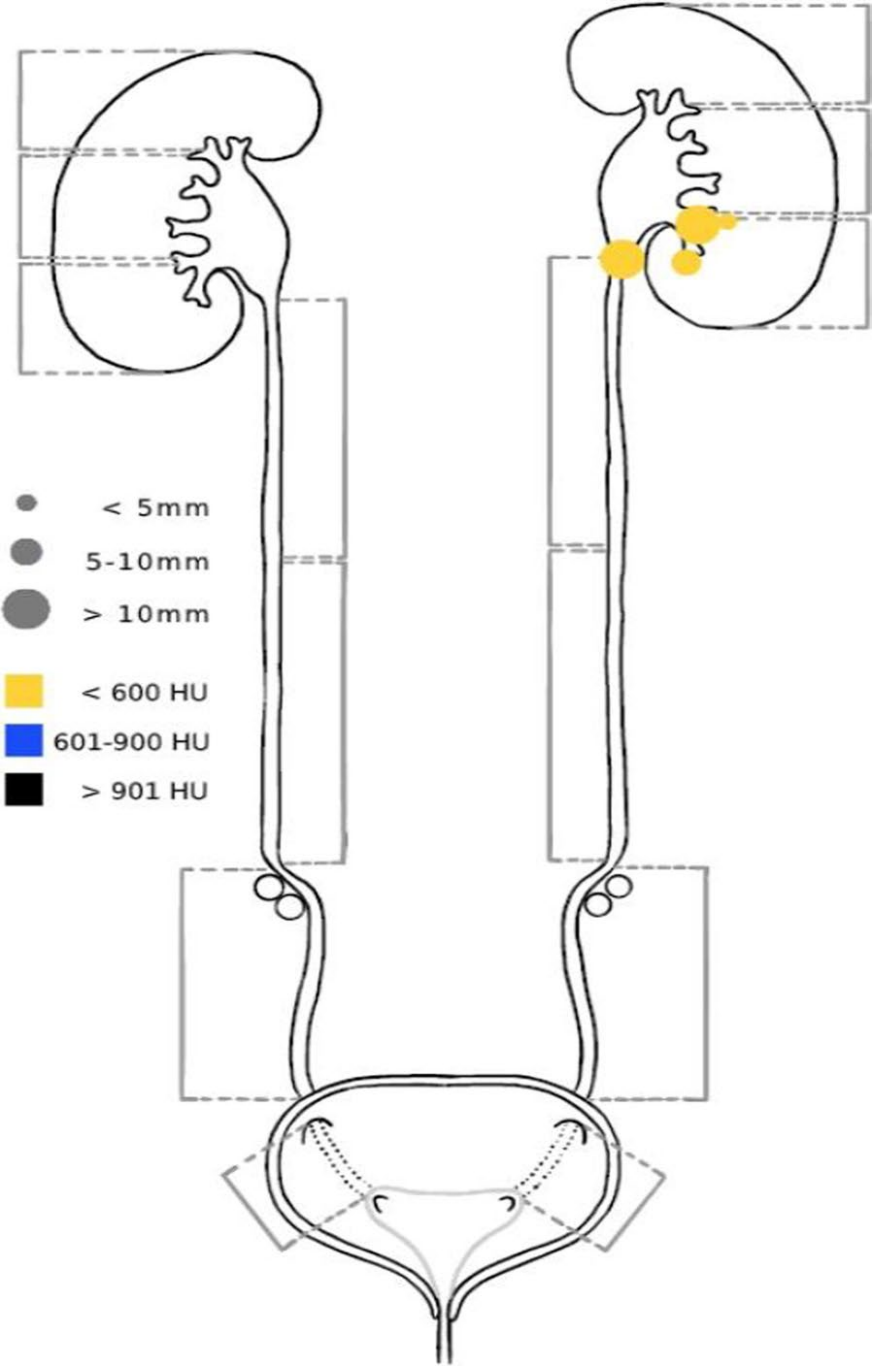
Graphic visualisation aid for urolithiasis CT. In the shown case, the radiologist has highlighted four stones of different sizes and low density in the left kidney and left proximal ureter

At our institution, an IHE MRRT–compliant web-based platform for SR was first implemented in 2016 [20]. Since then, several SR templates for various examinations in different imaging modalities have been created and have been established in clinical routine. The content of our SR templates is oriented on the basis of proposals for SR by the German Society of Radiology (*Deutsche Röntgengesellschaft*) and the RSNA RadReport library, which are both freely available online [11, 21]. Being in html form, our reporting templates are easily adjustable. All reports are stored in a database, which makes versatile evaluations feasible. The reporting templates for cardiac CT, urolithiasis CT, CT-A of the lower extremities, and prostate MRI were enhanced with interactive multimedia reporting features. For example, our urolithiasis template offers a graphical representation of the urinary tract, in which radiologists can highlight renal and ureteral calculi in different colours and dimensions according to their density and size (Fig. 1). The figures are automatically sent to the picture archiving and communication system (PACS) as a digital imaging and communications in medicine (DICOM) secondary capture along with the final report. The template development is an iterative process which also involves clinical referrers in order to tailor templates to their needs. Meetings of an expert group for SR consisting of consultants, residents, and IT specialists are held weekly. Their goal is the maintenance and adaptation of existing SR templates as well as the development of new ones. SR usage is encouraged and recommended at our institution, but not mandatory.

### Evaluation of the SR database

We queried the SR platform for all frequently used SR templates. For the ten most frequently used templates, the number of reports stored in our reporting platform was calculated on a quarterly basis. Between January 2016 and December 2022, the total number of reports for these examinations (the sum of structured reports and free-text reports) was queried from the RIS (Mesalvo RadCentre, Mannheim, Germany). We analysed the proportions of free-text reports and structured reports on a quarterly basis. The number of free-text reports was calculated by subtracting the number of structured reports from the total number of radiological reports. We also calculated annual proportion of SR for the following examinations: trauma CT, pulmonary embolism CT, focussed assessment with ultrasound in trauma (FAST), PET/CT, carotid ultrasound, and prostate MRI from 2016 to 2022. The proportion of reports using SR was also calculated for the imaging modalities CT, MRI, and ultrasound.

### Survey

An online survey (www.soscisurvey.de) was conducted to ask radiologists and referring physicians (urologists, trauma surgeons, orthopaedic surgeons, and doctors from internal medicine) for their opinions on forms of radiology reports.

A cognitive pre-test was performed with three final-year medical students and a board-certified radiologist to ensure the comprehensibility of the questionnaire. After minor reformulations, a standard pre-test was performed. Standard pre-testing led to technical modifications to ensure optimal visualisation on mobile phones.

The link to the finalised survey was e-mailed to the consultants of the above-mentioned departments with the request to forward it to all doctors in their departments and to all doctors in the radiology department.

The survey took place from August to September 2021 and was conducted in the German language.

First, the participants were surveyed regarding their gender, age, professional experience, specialisation, and board certification.

Second, they were asked about the characteristics of free-text reports and structured reports (“…are complete”; “…are clear”; “…enable fast extraction of relevant information”; “…facilitate decision-making”; “…enable research”) and to evaluate statements concerning reporting (“findings sections should be structured”; “impression sections should be structured”; “graphics and/or images embedded in structured reports would help to visualise findings/results”; “besides structured content, content in unstructured form will always be necessary”; “it is not possible to use SR for each examination”). Participants rated their agreement with these statements, using a 7-point scale (“do not agree at all”; “disagree”; “rather disagree”; “neutral”; “rather agree”; “agree”; “totally agree”). A 7-point scale leads to high variance among answers and thus to higher reliability and more nuanced trend analysis [22].

Lastly, the participants were queried about the optimal proportional use of structured reports and free-text reports with a slider indicating percentages.

### Statistical analysis

For statistical analysis, database entries were extracted as a CSV file and subsequently analysed using Excel 2016 (Microsoft, Redmond, USA). Results are reported in absolute numbers and as percentages as mentioned above.

Final survey results were extracted as a CSV file and subsequently analysed using R 4.0.3 (R Foundation for Statistical Computing, Vienna, Austria). Mean values and standard deviation values were calculated to analyse the survey results. Data distribution was tested using the Shapiro–Wilk test. As the variables were not normally distributed, the non-parametric Mann–Whitney U test was used to identify differences. A *p* value < 0.05 was considered statistically significant.

## Results

### Evaluation of the SR database

As of December 2022, our reporting platform contained 22,902 structured reports. The ten most frequently used templates accounted for 20,932 (91% of all) reports. Table 1 lists these ten templates with the total number of reports and the dates of implementation into the clinical workflow. The liver transplant evaluation template was the first to be established, in Q2 2016. For this template, we successfully implemented electronic report sheets that are required by German law for organ transplantation in clinical routine [23].

**Table 1:** Total number of structured reports for each of the ten most frequently used templates, listed by total number of reports

SR template	Total number of reports	Implemented in clinical routine since
PET-CT	4819	Q1 in 2017
Urolithiasis CT	3086	Q3 in 2018
FAST (focussed assessment with sonography in trauma)	2964	Q1 in 2018
Carotid duplex ultrasound	2587	Q4 in 2016
Pulmonary embolism CT	2366	Q4 in 2018
Trauma CT (whole body)	1646	Q3 in 2016
Cardiac CT	1243	Q1 in 2020
Prostate MRI	1131	Q1 in 2020
ABI (ankle brachial index) ultrasound	600	Q4 in 2017
Liver transplant evaluation (CT and MRI)	490	Q2 in 2016

The most frequently used SR template at our institution is for PET-CT, followed by those for urolithiasis CT and focussed assessment with sonography in trauma (FAST). The latest SR templates to be implemented are for CT-angiography of the lower extremities, in Q2 2022, and for pre-procedural CT for transcatheter aortic valve replacement, in Q3 2022.

**Fig. 2:**
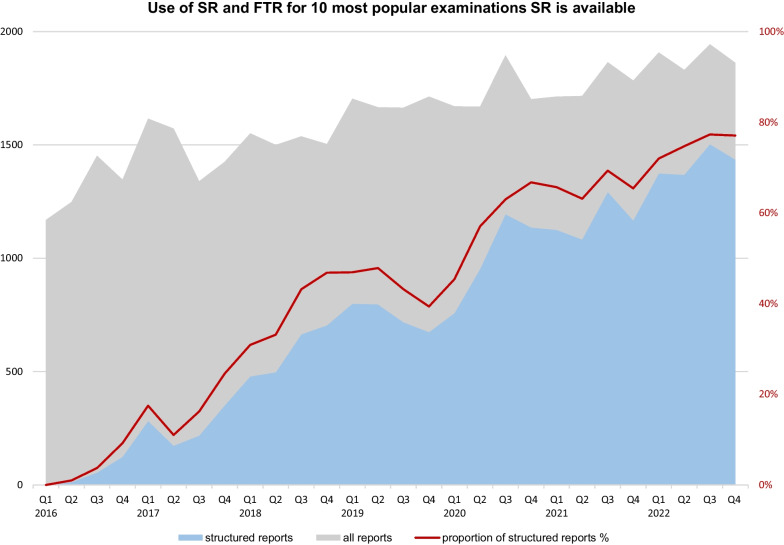
Absolute numbers of all radiological reports (grey) and structured reports (blue) as well as the proportion of structured reports (red) over a 7-year period on a quarterly basis for the ten most frequently used templates

Quarterly analysis of the proportions of structured reports and free-text reports for these ten examination types for which SR is most used showed a steady increase of SR usage up to 77% in Q4 2022, while the total numbers for these examination types showed an increase of 49%, from 1248 in Q2 2016 to 1862 in Q4 2022 (Fig. 2).

**Fig. 3:**
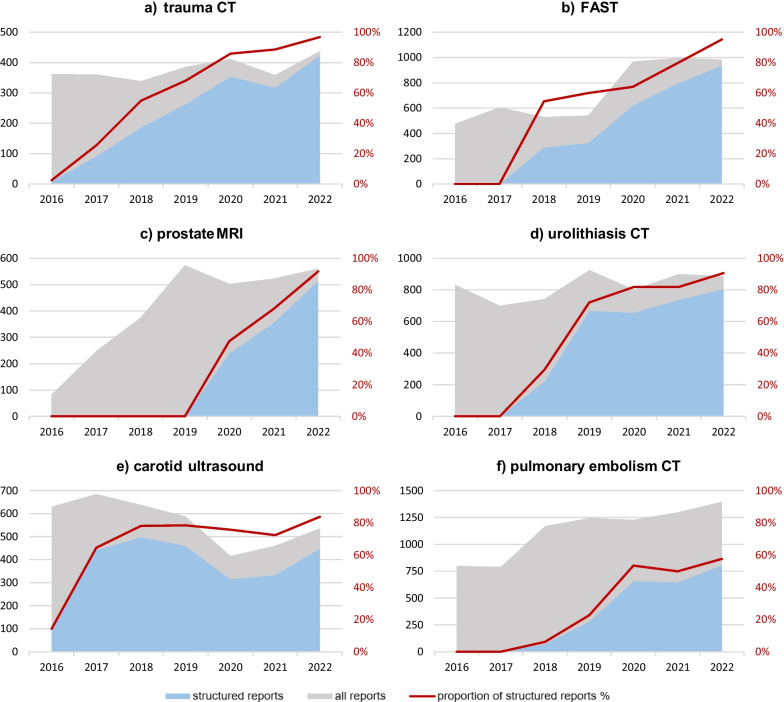
Absolute numbers of all radiological reports (grey) and structured reports (blue) as well as the proportion of structured reports (red) over a 7-year period, shown for (**a**) trauma CT, (**b**) focussed assessment with ultrasound in trauma (FAST), (**c**) prostate MRI, (**d**) urolithiasis CT, (**e**) carotid ultrasound, and (**f**) pulmonary embolism CT

Further analysis of single examinations showed that in trauma CT, FAST, and prostate MRI, SR usage was the highest (97%, 95%, and 92%, respectively, in 2022), followed by urolithiasis CT and carotid ultrasound with SR use at 91% and 84%, respectively (Fig. 3). In pulmonary embolism CTs, some radiologists still tend to report in a free-text form (SR usage 58% in 2022). For all examinations, an increase in the use of structured reports since their implementation was shown. Liver transplant evaluation was excluded from the analysis, since it is required to be in a structured form by German law (SR usage 100%).

**Fig. 4:**
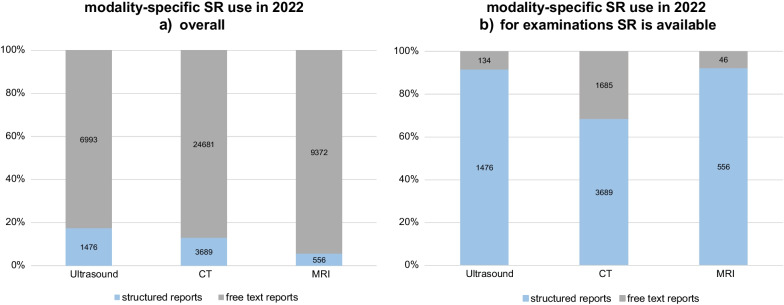
Modality-specific use of SR in 2022: (**a**) for all examinations, and (**b**) for those for which SR was available, with absolute numbers for free-text reports and structured reports

Analysis of annual modality-specific use of SR showed increasing values for CT, MRI, and ultrasound. Figure 4 shows the modality-specific use of SR at the end of the period under review (2022), on the one hand for all examinations carried out in each modality (A) and on the other hand for all examinations for which SR was available (B). Overall, SR was used in reporting ultrasound, CT, and MRI in 17%, 13%, and 6% of reports, respectively. Regarding only examination types for which SR was available, modality-specific SR use was much higher, at 92%, 69%, and 92% for ultrasound, CT, and MRI, respectively. No use proportions for x-ray examinations, mammographies, and angiographic procedures were calculated since, as yet, no SR templates have been developed for any examinations in these modalities at our institution.

### Survey results

A total of 51 physicians (15 radiologists and 36 referring physicians) from our institution took part in the survey. The study participants’ demographic information is shown in Table 2. The mean professional experience of the participants was 9 years in both groups. The mean age of radiologists was 36 years and mean age of referring physicians was 37 years. In both groups, residents and board-certified physicians took part in the survey.

**Table 2:** Demographic information collected in this survey. Total numbers or mean ± standard deviation

	Radiologists (n = 15)	Referring physicians (n = 36)
Gender (female)	40%	30%
Age (years)	36 ± 12	37 ± 7
Professional experience (years)	9 ± 12	9 ± 7
Board certification	47%	53%

**Fig. 5:**
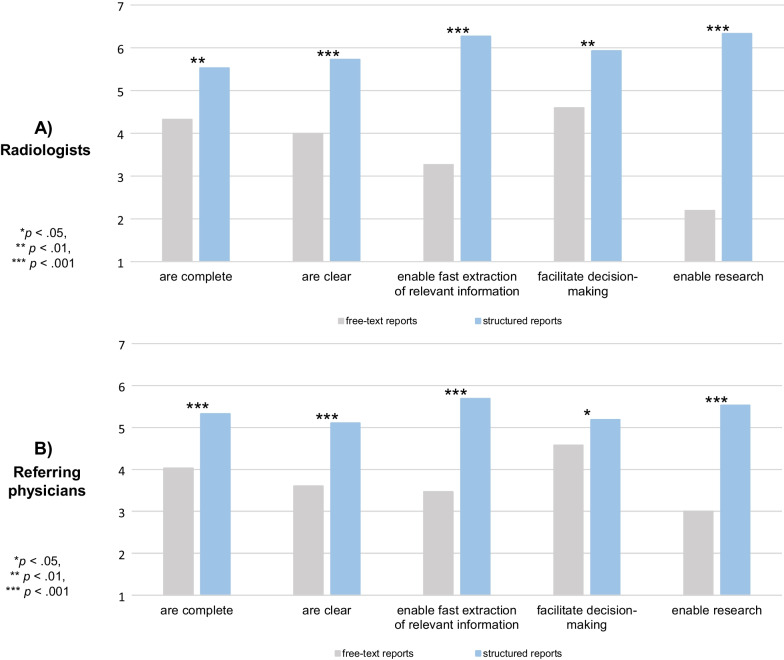
Subjective evaluation of structured reports and free-text reports by radiologists (**A**) and referring physicians (**B**) on a 7-point scale. Grey bars represent free-text reports and blue bars structured reports

Radiologists and referring physicians both rated structured reports as significantly better than free-text reports for the following attributes: completeness, clarity, enabling fast extraction of relevant information, facilitating decision-making, and enabling research (Fig. 5).

Radiologists and referring physicians agreed that the findings section of reports, especially, should be structured. Nevertheless, they stated that unstructured content will always be necessary in radiological reports and that structured reporting will not be possible for every indication. Radiologists agreed more with the statement that graphics and/or images embedded in a structured report help to visualise the imaging results (*p* = 0.003). Table 3 shows the ratings of all statements by radiologists and referring physicians.

**Table 3:** Ratings of the statements (referring physicians vs. radiologists, 7-point- scale)

Statement	Radiologist	Referring physician	*p* value
Findings sections should be structured	6.07 ± 0.80	5.97 ± 0.81	0.780
Impression sections should be structured	5.20 ± 1.37	5.17 ± 1.65	0.874
Graphics and/or images embedded in structured reports help to visualise findings/results	6.40 ± 0.74	5.44 ± 1.23	0.003
Besides structured content, content in unstructured form will always be necessary	5.93 ± 1.22	5.31 ± 1.37	0.126
It is not possible to use SRs for every examination	5.60 ± 1.64	5.00 ± 1.64	0.201

**Fig. 6:**
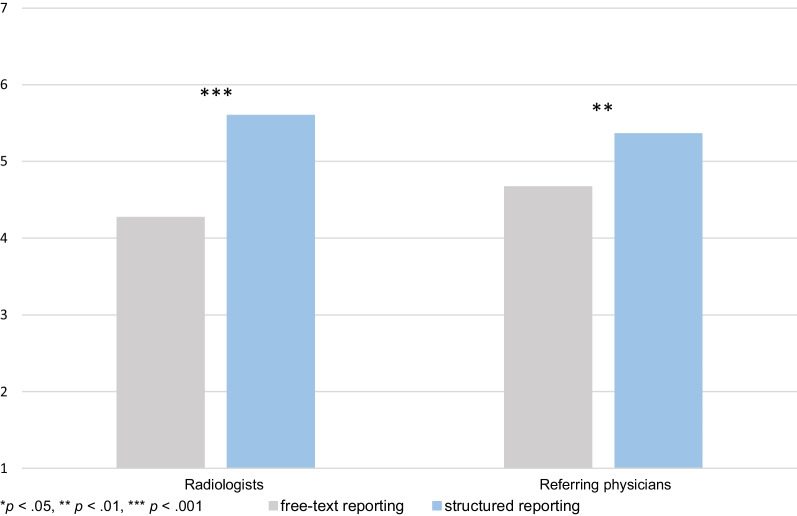
Satisfaction of referring physicians with SR and FTR predicted by radiologists (left) and self-reported satisfaction of referring physicians with SR and FTR (right)

Radiologists predicted that SR leads to a significantly higher referring-physician satisfaction compared with FTR. Referring physicians reported themselves to be more satisfied with SR than with FTR (Fig. 6).

We did not find a significant difference between radiologists’ and referring physicians’ views regarding the optimal proportions of free-text and structured reports (*p* = 0.64). Radiologists estimated the respective optimums as 32% and 68%, and referring physicians as 36% and 64%.

## Discussion

In this study, we evaluated the clinical implementation of SR at our institution over the last 7 years. A steadily increasing total number of structured reports as well as an increasing percentage of SR in radiology reports could be observed. In the survey, radiologists and referring physicians both agreed on many advantages of SR over free-text reporting. The data show that SR implementation is a long-term process requiring great effort but that it is ultimately successful in terms of SR usage in clinical routine.

Explicit data on actual rates of SR usage in clinical routine are very limited. Published surveys on SR usage deal mostly with the question of how many institutions are or are not using SR in clinical routine, but do not reveal specific use ratios [19, 24]. A few older publications on successful implementations at U.S. institutions do describe high SR usage and compliance among radiologists [18, 25, 26]. Nevertheless, their definition of SR would be classified as standardised reporting according to the current state of knowledge and does not refer to an IT-based method [7].

For those examinations for which SR templates are available, we demonstrated high proportions of SR use of up to 97% (trauma CT template in 2022), even though SR use is not mandatory at our institution. This confirms the growing acceptance of SR among radiologists, which is further underlined by the positive attitude towards SR revealed in the survey part of this study. The positive results of our survey highlight the success of the implementation process and coincide with other surveys that have evaluated SR for examinations including pulmonary embolism CT, trauma CT, and many others [3, 5, 6]. Nevertheless, these previous surveys dealt with out-of-hospital evaluations to determine the value of SR for particular examinations and had mostly small numbers of participants. Our survey, on the other hand, was conducted during an actual clinical implementation of SR for many examinations and had a larger participant count (51) than the prior ones.

Differences in the use of SR between single examination types, such as the high rate seen in prostate MRI compared with the relatively lower rate in pulmonary embolism CT (92% vs. 58% in 2022) may be due to the nature of the examinations. After all, the implementation of SR is highly case specific [12]. Our prostate MRI template offers decision support on how to classify lesions according to PI-RADS and contains a map of the prostate sectors as an interactive multimedia reporting feature. Users are guided through the report, which makes SR the favoured form of reporting for most of them. In pulmonary embolism CT, findings that point toward an alternative diagnosis are found in up to 33% of the examinations [27]. Radiologists might find the template unsuitable for properly describing alternative diagnoses.

The need for interactive multimedia reporting features like graphical visual aids is addressed by various publications [10, 28]. Some of our templates are supported by those features, and commercial vendors have already started integrating them into their SR solutions. To date, no investigations have objectively assessed their benefits. In our survey, radiologists and referrers agreed that they are helpful. Especially in interdisciplinary meetings, graphic visual aids have the potential to accelerate and facilitate workflow and communication [29].

Obstacles in the way of successfully implementing SR in clinical routine have been discussed in the literature and are mostly of an organisational, technical, or personal nature [15, 16, 18]. Organisational difficulties could be mitigated by weekly expert meetings and the iterative form of template development.

A major technical obstacle is the limited integration of SR into the RIS. Our SR platform successfully communicates with the RIS via an HTTP and XML interface [20]. Since its implementation, various customisations of the RIS have been made in collaboration with the vendor, and these have further improved the integration of the reporting platform. For example, changes to the RIS user interface that facilitated access to the reporting templates were made, and loading times for templates could be reduced.

The data on high SR usage and the positive attitude towards SR indicate that personal aversions to SR among radiologists are low at our institution. On the contrary, radiologists desire more SR, and currently the limiting factor is the availability of templates. Over a 7-year process, radiologists have become accustomed to SR. Aversions from the early days might have faded and are addressed by subsequent teaching on how to use SR.

In order to reach higher overall SR use, the directed development of more templates is inevitable. Nevertheless, SR will probably not be applicable to all kinds of examinations. Radiologists and referring physicians agreed on this statement in the study survey and stated that the ratio between structured and free-text reports should be approximately 2:1.

SR is not equally suitable for all examinations, and a practical application to every examination might not be possible at all. Nevertheless, the estimated equilibrium should be reachable in the near future. Therefore, the few remaining disadvantages of SR have to be resolved. For example, natural language processing has the potential to integrate speech recognition into SR and thus could further boost SR usage.

Our study has several limitations. It was conducted as a single-centre study and therefore carries associated risks, such as potentially limited external validity [30]. Most other institutions that work with an IHE MRRT-compliant reporting platform do not have a comparably large number of structured reports in their databases, which makes detailed validation difficult. Moreover, we ourselves chose the examinations for which we would develop SR templates. This might carry a slight selection bias, since SR usage might be more feasible for the chosen examinations than for others.

## Conclusion

The data on high SR usage along with the positive attitudes of both radiologists and clinical referrers towards SR shows that the clinical implementation of SR can be successful. We therefore strongly encourage others to take this step. Ultimately, the added value that SR provides is definitely worth the effort.

## Data Availability

The datasets used and/or analysed during the current study are available from the corresponding author on reasonable request.

## Abbreviations

CT: Computed tomography
DICOM: Digital imaging and communications in medicine
FAST: Focussed assessment with ultrasound in trauma
FTR: Free-text reporting
IHE MRRT: Integrating the Healthcare Enterprise Management of Radiology Report Templates
IT: Information technology
MRI: Magnetic resonance imaging
PACS: Picture archiving and communication system
PET: Positron emission tomography
PI-RADS: Prostate imaging reporting and data system
RIS: Radiology information system
SR: Structured reporting
